# Radiographic acceptable zone of endobutton placement in ACL reconstruction: A prospective study

**DOI:** 10.1002/jeo2.70082

**Published:** 2024-11-20

**Authors:** Arash Sharafatvaziri, Mohammad Tahami, Maryam Salimi, Hamid Rabie, Fardis Vosoughi, Morad Karimpour, Ghazaleh Moradkhani, Mosayeb Soleymani

**Affiliations:** ^1^ Department of Orthopedic Surgery Shariati Hospital Tehran Iran; ^2^ Center for Orthopedic Trans‐Disciplinary Applied Research Tehran University of Medical Sciences Tehran Iran; ^3^ Department of Orthopedic Surgery Chamran Hospital Shiraz Iran; ^4^ Department of Orthopedic Surgery School of Medicine, Chamran Hospital Shiraz University of Medical Sciences Shiraz Iran; ^5^ Department of Orthopedic Surgery School of Medicine, Shariati Hospital Tehran University of Medical Sciences Tehran Iran; ^6^ Department of Mechanical Engineering College of Engineering, University of Tehran Tehran Iran

**Keywords:** ACL reconstruction, arthroscopy, endobutton, femoral tunnel, plain radiograph

## Abstract

**Purpose:**

During the transportal technique of anterior cruciate ligament (ACL) reconstruction, tunnel outlet location can be varied depending on certain anatomical and technical characteristics. Therefore, we aimed to find out the acceptable zone of endobutton location by introducing several radiographic values.

**Methods:**

Postoperative lateral radiographs of 72 patients were assessed to measure the distances from the centre of the button to the posterior femoral cortex (D1) and to the most distal point of the lateral condyle (D2). Furthermore, based on the anteroposterior (AP) radiographs, the distances from the centre of the button to the lateral femoral cortex (D3) and from the centre of the button to the line connecting the most distal points of the medial and lateral femoral condyles (D4) were assessed. To measure the sensitivity and specificity of each radiographic value (D1, D2, D3 and D4), the area under the receiver operating characteristic curve was calculated. The alpha angle and femoral tunnel length values were considered as gold standards.

**Results:**

Analyses showed that the mean values for D1, D2, D3 and D4 were 13.20 ± 0.54, 39.44 ± 0.31, 1.65 ± 0.15 and 42.66 ± 0.47 mm, respectively. The mean angle was found to be 38.6 ± 0.3°, and the mean femoral tunnel length was 38.6 ± 0.2 mm. Age was significantly related to D2 and the diameter of the femur in AP X‐ray, while body mass index had a significant relation with D3 (*p* < 0.05).

**Conclusion:**

In this study, a new method was proposed to evaluate the accuracy of anatomical tunnel placement in ACL reconstruction surgery postoperatively. The statistical analysis of the measured variables showed that the mean ratios were 21.79 ± 0.87 for D1, 65.65 ± 0.63 for D2 and 51.90 ± 0.73 for D4. The results indicated that if the tunnel exit location and endobutton placement in the postoperative radiological images fall within the suggested areas, it can be meaningfully concluded that the tunnel is correctly positioned intraarticularly and the ligament reconstruction is anatomical.

**Level of Evidence:**

Level III.

**Abbreviations**
ACL: anterior cruciate ligament
AP: anteroposterior
AUC: area under the curve
BMI: body mass index
FD. L: femoral diameter in lateral X‐ray
FD.AP: femoral diameter in AP X‐ray

## INTRODUCTION

Anterior cruciate ligament (ACL) tear is one of the most common ligamentous injuries, particularly among young people and athletes. Several methods exist for ACL reconstruction, with the flipping mechanism using femoral cortical suspension devices, such as the EndoButton® CL (Smith & Nephew), being recognised as a reliable and effective solution [13]. One of the main factors determining the success and prognosis in this surgery is choosing the right location of the femoral tunnel. Although the location of endobutton is not a comprehensive representative of femoral tunnel location and orientation, many surgeons continue to evaluate the location of the tunnel outlet to assess the correctness of femoral tunnel placement. Postoperative standard knee radiograph is the common method for assessing the location of endobutton as an indicator of tunnel outlet [5]. Therefore, various anatomical parameters have been suggested as ideal locations for endobutton placement. These include the femoral angle, endobutton position and the relationship of the tunnel to the line drawn along the intercondylar notch roof (Blumensaat's line) [1].

However, in some surgical techniques, especially independent femoral drilling methods such as transportal techniques, canal length, obliquity and tunnel outlet, location can vary depending on certain anatomical and technical characteristics [10]. Thus, routine measurement of radiological parameters neither confirm nor deny the correct location of the tunnel.

Many surgeons evaluate the location of endobutton, representative of tunnel outlet, to assess the correctness of femoral tunnel placement. The aim of this study is to provide a solution for validating ACL reconstruction using endobutton placement in patients who underwent ACL reconstruction via the transportal method with a standard femoral tunnel entry point. This validation will be achieved by analysing postoperative radiological images. In other words, using this method, if the surgeon has reconstructed the ACL in the anatomical femoral position and the tunnel exit and endobutton position in the postoperative radiological images fall within the suggested range of this study, it can be concluded that the reconstruction was performed anatomically.

## MATERIALS AND METHODS

### Study design and participant selection

This prospective study, conducted from February 2020 to March 2021, included 72 patients diagnosed clinically and radiologically with a complete isolated primary ACL tear. The patients, aged between 18 and 45 years with a mean age of 28.6 ± 0.5 years, underwent arthroscopic transportal single‐bundle ACL reconstruction using an autologous quadruple hamstring tendon graft. Demographic data collected for each patient included age, sex, body mass index (BMI) and the injured side. The mean BMI of the patients was 24.3 ± 0.3 kg/m^2^.

### Surgical procedure

Our standard arthroscopic ACL reconstruction protocol involved an initial diagnostic knee arthroscopy to evaluate and address any associated pathologies and to confirm the severity of the ACL rupture. Subsequently, the semitendinosus tendon, with or without the gracilis tendon, was harvested based on the size of the ACL footprint. ACL remnants were debrided using a motorised shaver as necessary. In cases of a narrow notch, a lateral wall‐plasty or notchplasty was performed to prevent potential graft impingement on the lateral wall.

The femoral tunnel was typically created via a low anteromedial portal while viewing from a transpatellar portal. The centre of the femoral ACL footprint was marked with electrocautery. A suture eye guide pin was then placed at the centre of the ACL femoral footprint to serve as an aiming guide for the femoral reamer (ConMed, Linvatec). Variations in the orientation and outlet of the femoral tunnel can be influenced by the knee flexion angle and the drilling angle. To minimise these confounding factors, the procedure was performed with the knee in maximal flexion and a drilling angle of 40°.

**Figure 1:**
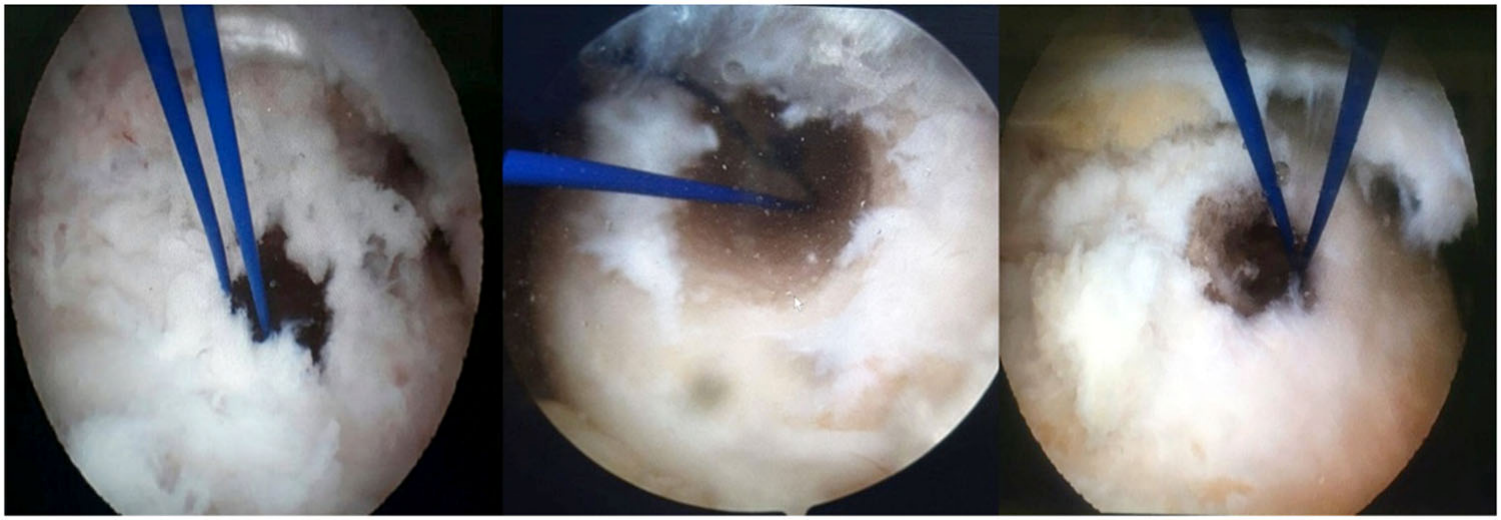
Arthroscopic images of the femoral tunnel.

Initially, the footprint was reamed using a 4.5 mm reamer all the way to the lateral cortex to facilitate the future passage of the endobutton. The femoral tunnel was then reamed to match the size of the prepared graft, typically between 20 and 25 mm. To document the anatomical location of the femoral tunnel, a photograph of the arthroscopic view was taken at this stage of the procedure (Figure 1). These images were in JPEG format and were taken solely for research purposes.

The tibial tunnel was reamed using the ACL tibial guide (ConMed, Linvatec) at the footprint location, set at an angle of 55°. A looped nylon suture was then used to pass the GraftMax^®^ endobutton (ConMed, Linvatec) through both tunnels, followed by shuttling the graft with the adjustable strands of the endobutton. The graft was secured on the tibial side with an interference screw (ConMed, Linvatec).

### Clinical assessment and radiographic evaluations

The precise location of the femoral tunnel in each patient was documented by arthroscopic photography immediately after the surgery (Figure 1). Anteroposterior (AP) and lateral postoperative radiographs were obtained in a standard position, with the knee flexed at 15°. In the lateral radiographs, the knee joints were positioned in the centre of the cassette, with overlap of the medial and lateral femoral condyles. The patella was clearly visible with an unobstructed femoral space, and the platforms of the tibia and femur exhibited minimal overlap [18]. To eliminate measurement error, the radiological images were taken directly with no angulation to the knee. All radiographic images were in DICOM format, and measurements were performed by a single examiner blinded to the study, using a Windows‐based application (Marcopacs^®^). Additionally, to minimise individual measurement errors, two qualified individuals independently supervised the measurement process. These measurements included tunnel length and locations, as defined subsequently.

**Figure 2:**
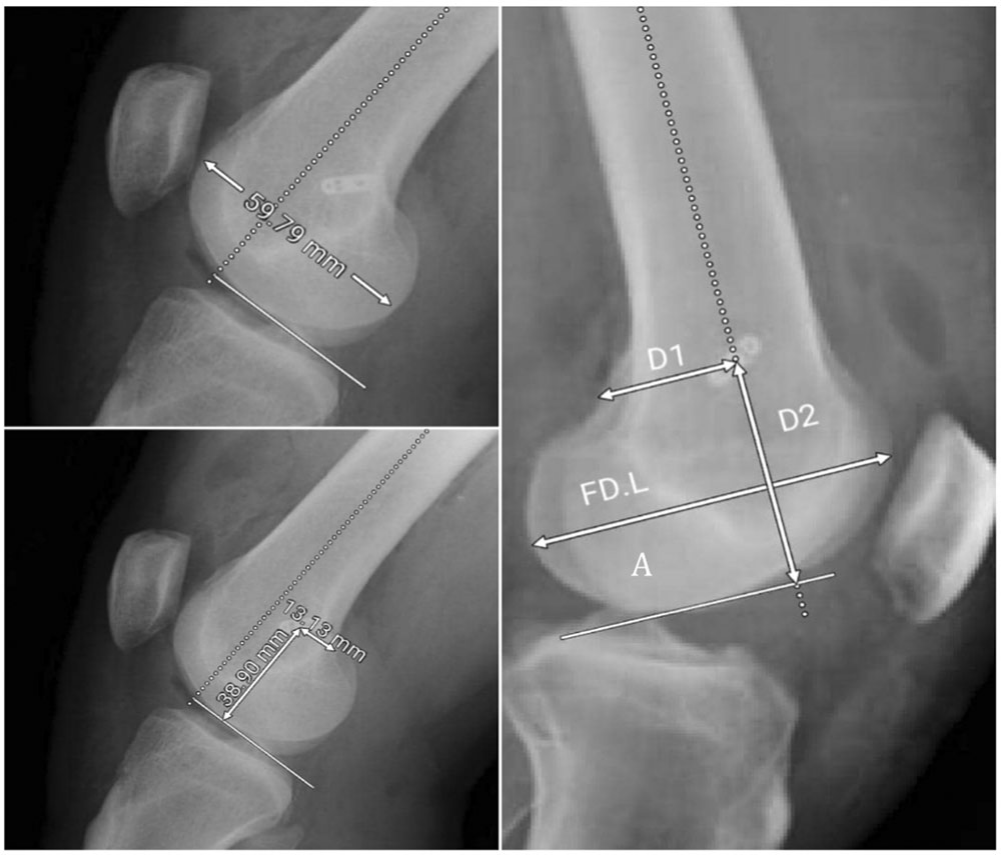
Features of lateral radiographs.

In the lateral radiographs, we specified several locations, including D1, D2 and FD.L (Figure 2). D1 was measured as the distance from the centre of the button to the posterior femoral cortex. It is parallel to line A, which is a line drawn vertically to the posterior femoral cortex at the most distal point of the lateral condyle. D2 is the distance from the centre of the button to line A. FD.L (femoral diameter in lateral X‐ray) was defined as the longest AP diameter of the femoral condyle. As the size of the knee affects measured distances, ratios were used to eliminate this factor by dividing each parameter by the FD.L.

**Figure 3:**
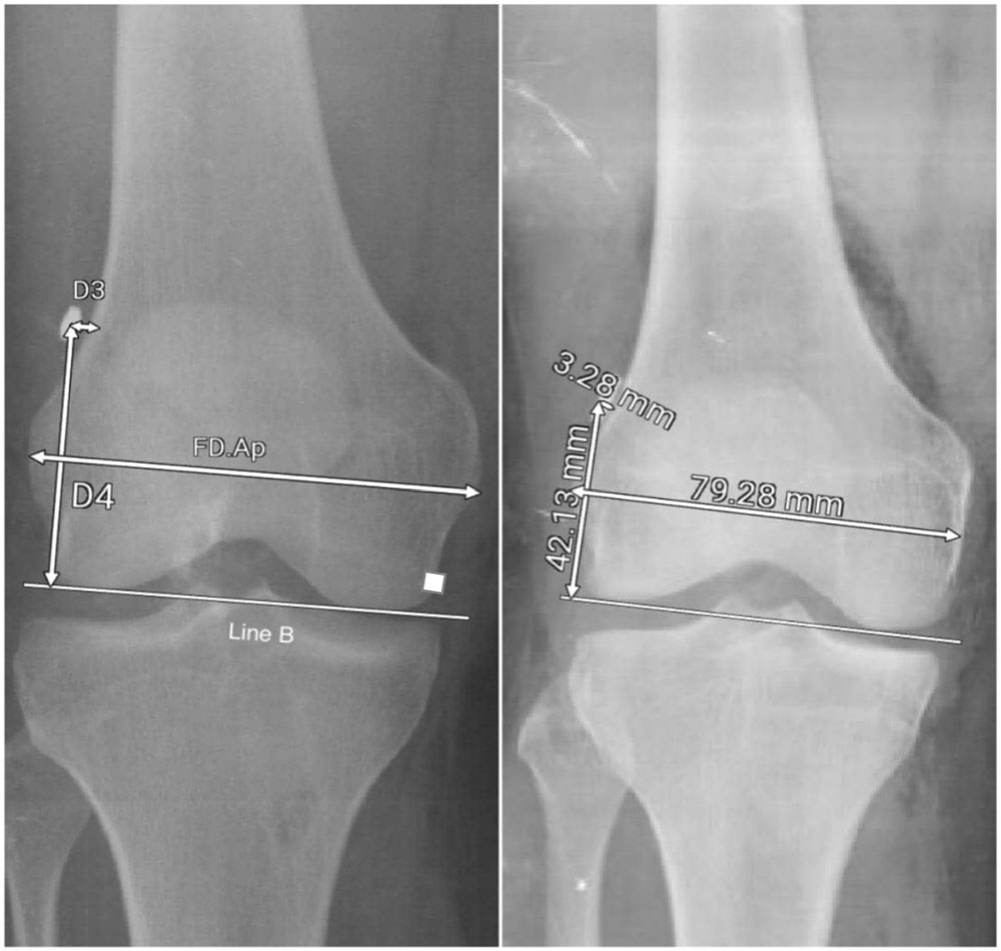
Features of anteroposterior radiographs.

**Figure 4:**
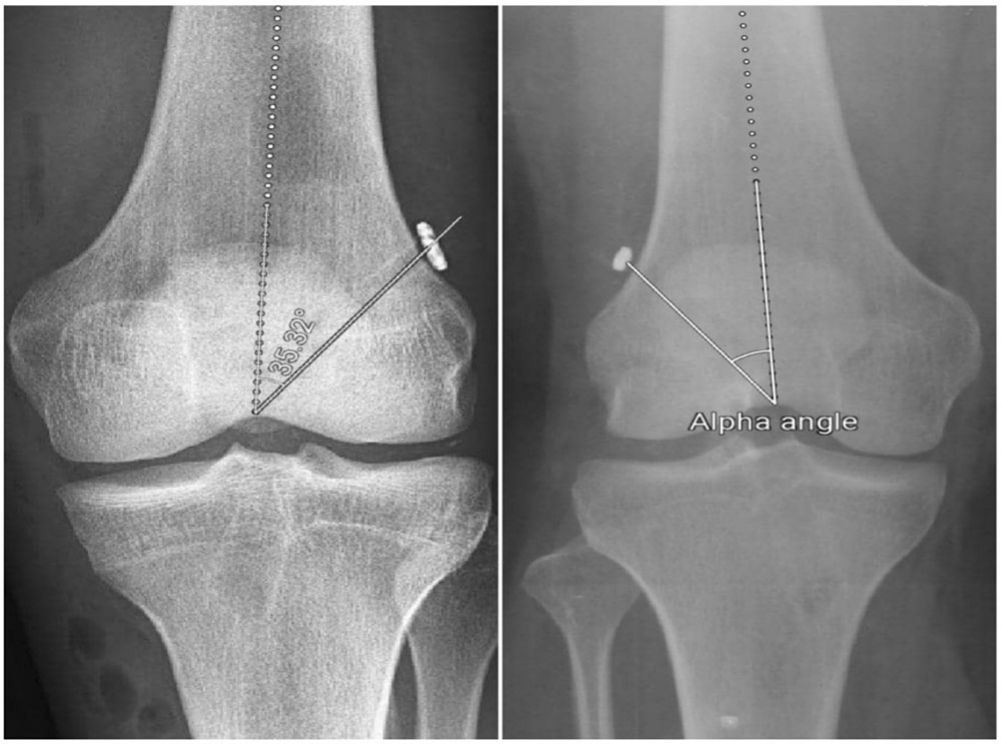
Alpha angle measured in anteroposterior radiographs.

In AP radiographs, D3 is measured as the distance from the button centre to the lateral femoral cortex. It is parallel to line B, which connects the most distal points of the medial and lateral femoral condyles. D4 is the distance from the button centre to line B. FD.AP (femoral diameter in AP X‐ray) is defined as the longest mediolateral diameter of the distal femur. To account for the knee size as a factor affecting the measured distances, each parameter was divided by the femoral diameter in the AP X‐ray (Figure 3). In the AP radiograph, the alpha angle was also measured, which is defined as the angle between the long axis of the femur and a line drawn from the centre of the button to the top of the intercondylar notch [7] (Figure 4).

### Statistical analysis

Statistical analysis was performed using SPSS version 23.0. The normal distribution of all data was assessed using the Kolmogorov–Smirnov test, and frequencies were calculated for all variables. Independent samples *t* test, Mann–Whitney U test and Spearman's rank correlation coefficient were used to evaluate comparisons between variables. Correlations were considered significant if they had a *p *< 0.05.

After the initial analysis, the sensitivity and specificity of each radiographic value (D1, D2, D3 and D4) were measured by calculating the area under the curve (AUC). The angle and femoral tunnel length values were considered gold standards, based on the mean angle and femoral tunnel length reported in studies by Illingworth et al. [7] and Iyyampillai et al. [8], respectively.

### Ethical consideration

This study was conducted in accordance with the seventh revision of the Helsinki Declaration. All participants provided written informed consent. Efforts were made to ensure the rigorous anonymity of their records. Furthermore, the study was approved by the ethical committee of the Centre for Orthopaedic Transdisciplinary Applied Research (COTAR) (IRB number: IRB00007932).

## RESULTS

The present study was conducted on 72 patients (seven females and 65 males) between February 2020 and March 2021. The ACL tear was present in 31 right knees and 41 left knees. During surgery, 30 patients had a medial meniscus tear and 20 had a lateral meniscus tear. Among those with a medial meniscus tear, 10 patients underwent meniscal repair and 15 underwent meniscectomy. Among those with a lateral meniscus tear, eight patients underwent meniscal repair and 10 underwent meniscectomy.

Measurement analysis showed D1 with the total mean of 13.2 ± 0.5 mm, D2 with 39.4 ± 0.3 mm, FD.L with 59.5 ± 0.4 mm, D3 with 1.6 ± 0.1 mm, D4 with 42.6 ± 0.4 mm and FD.AP with 81.4 ± 0.6 mm. The mean degree of alpha angle was found to be 38.6 ± 0.3, and the mean femoral tunnel length was 38.6 ± 0.2 mm. The ratio of each parameter (D1 to D4) was also calculated by dividing the parameter by the FD‐AP and FD‐L. No significant relationship was found between the radiologic parameters and gender (Table 1).

**Table 1:** Demographic data and radiologic features by gender.

**Variables;** mean ± SD	**Gender**			***p* Value**
	**Male** *n* = 65	**Female** *n* = 7	**Total** *n* = 72	
Age	28.86 ± 0.56	26.71 ± 1.53	28.65 ± 0.53	0.235
BMI	24.33 ± 0.34	24.28 ± 0.68	24.33 ± 0.31	0.961
D1	13.35 ± 0.56	11.85 ± 2.17	13.20 ± 0.54	0.424
D2	39.44 ± 0.32	39.42 ± 1.36	39.44 ± 0.31	0.987
FD. L	59.66 ± 0.45	58.85 ± 0.96	59.58 ± 0.41	0.572
D3	1.67 ± 0.16	1.42 ± 0.52	1.65 ± 0.15	0.643
D4	42.66 ± 0.51	42.71 ± 0.91	42.66 ± 0.47	0.974
FD.AP	81.50 ± 066	81.00 ± 2.05	81.45 ± 0.62	0.239
D1 ratio	22.03 ± 088	19.57 ± 3.66	21.79 ± 0.87	0.407
D2 ratio	65.61 ± 0.67	66.00 ± 2.00	65.65 ± 0.63	0.858
D3 ratio	1.86 ± 0.18	1.85 ± 0.63	1.86 ± 0.17	0.994
D4 ratio	51.69 ± 0.80	52.85 ± 1.03	51.90 ± 0.73	0.386
Alpha angle	38.63 ± 0.34	38.85 ± 1.31	38.65 ± 0.33	0.842
Tunnel length	38.73 ± 0.24	37.71 ± 0.71	38.63 ± 0.23	0.194

Abbreviations: BMI, body mass index; FD.AP, femoral diameter in anteroposterior X‐ray; FD.L, femoral diameter in lateral X‐ray; SD, standard deviation.

It was found that age was significantly related to D2 (*p* = 0.044) and FD.AP (*p* = 0.013). The correlation coefficients indicated that both D2 and FD.AP have a weak positive relationship with age. BMI was also significantly related to D3 (*p* = 0.005), with this correlation also being weak and positive. The correlation coefficients between age, BMI and the radiographic parameters are shown in Table 2.

**Table 2:** Radiologic features by age and BMI.

**Radiographic parameters**	**Age**		**BMI**	
	**Correlation coefficient**	***p* Value**	**Correlation coefficient**	***p* Value**
D1	0.04	0.741	−0.212	0.073
D2	0.238	0.044*	0.124	0.299
FD. L	−0.47	0.698	0.003	0.977
D3	−0.019	0.877	0.329	**0.005** *
D4	−0.020	0.866	0.154	0.195
FD.AP	0.290	0.013*	−0.107	0.371
Alpha angle	0.132	0.268	−0.120	0.317
Tunnel length	−0.163	0.171	−0.002	0.987

Abbreviations: BMI, body mass index; FD.AP, femoral diameter in anteroposterior X‐ray; FD.L, femoral diameter in lateral X‐ray.

* *p* < 0.05 (significant).

Interestingly, the affected side was not significantly related to any of the measured values (Table 3).

**Table 3:** Radiologic features by affected side.

Variables; mean ± SD	Affected side		*p* Value	95% CI
	Right side *n* = 31	Left side *n* = 41		
D1	13.38 ± 0.6	13.07 ± 0.8	0.846	12.112–14.304
D2	39.74 ± 0.5	39.21 ± 0.4	0.350	38.817–40.071
FD.L	60.29 ± 0.6	59.04 ± 0.5	0.174	58.749–60.416
D3	1.93 ± 0.2	1.43 ± 0.2	0.126	1.339–1.966
D4	42.45 ± 4.07	42.82 ± 3.9	0.695	41.725–43.608
FD.AP	81.64 ± 0.9	81.31 ± 0.8	0.882	80.211–82.705
Alpha angle	38.38 ± 0.5	38.85 ± 0.4	0.544	37.987–39.317
Tunnel length	38.80 ± 0.3	38.51 ± 0.3	0.594	38.175–39.102

Abbreviations: CI, confidence interval; FD.AP, femoral diameter in anteroposterior X‐ray; FD.L, femoral diameter in lateral X‐ray.

Results of the AUC, specificity and sensitivity factors are demonstrated in Tables 4 and 5.

**Table 4:** Area under the curve of radiographic values based on angle and tunnel.

**Values**	**Area**		**95% CI**		***p* Value**		**Cut‐off (associated criterion)**	
	**Angle**	**Tunnel**	**Angle**	**Tunnel**	**Angle**	**Tunnel**	**Angle**	**Tunnel**
D1	0.763	0.740	0.648–0.855	0.623–0.836	0.033	0.032	>7	≤20
D2	0.530	0.604	0.409–0.649	0.481–0.717	0.782	0.215	≤37	≤39
D3	0.510	0.545	0.390–0.630	0.424–0.663	0.945	0.741	>0	≤2
D4	0.519	0.558	0.398–0.639	0.436–0.675	0.883	0.618	>42	≤39

Abbreviation: CI, confidence interval.

**Table 5:** Sensitivity and specificity of radiographic values based on reference ranges of angle and tunnel.

**Values**	**Angle**		**Tunnel**	
	**Sensitivity**	**Specificity**	**Sensitivity**	**Specificity**
D1	88.06	60.00	95.45	50.00
D2	25.37	100.00	54.55	83.33
D3	73.13	40.00	69.70	50.00
D4	49.25	80.00	24.24	100.00

## DISCUSSION

In this study, a new method was proposed to evaluate the accuracy of anatomical tunnel placement in ACL reconstruction surgery postoperatively. Despite the high application of the transportal technique, there are still no standard values for assessing the correct location of the femoral tunnel in this technique through imaging. This study offers a method for validating ACL reconstruction through endobutton placement in patients who have undergone ACL reconstruction using the transportal technique with a standard femoral tunnel entry point. The validation is based on analysing postoperative radiological images. Essentially, if the surgeon has placed the ACL in the correct anatomical position and the tunnel exit and endobutton placement in the postoperative images align with the specified range of this study, it indicates that the reconstruction was performed accurately.

In the transportal technique, to create the femoral tunnel, the guidewire is first placed at the centre of the native ACL footprint. Then, with subject in maximal knee flexion, the femoral tunnel is drilled. Finally, the graft is fixed to the endobutton on the lateral cortex of the femur. However, sometimes the flexion angle may be reduced or increased during surgery, and as a result, although the entrance to the femoral tunnel can be adjusted, the location of the outlet may be different from the planned location [5]. Not only the flexion angle of the knee but also the angle of drilling can affect the variation in tunnel locations. According to previous studies, the optimum value for drilling angle is 30°–45° on the axial plane [7, 8]. We carried out the procedure at a 40° drilling angle and maximum knee flexion to rule out these interfering factors.

Unlike the transportal technique, the other two most popular techniques, the transtibial and the outside‐in drilling technique, do not show as much variation in femoral tunnel placement [4]. In the transtibial technique, which is the most traditional method, the tibial tunnel direction is the most important factor affecting the location of the femoral tunnel, rather than the degree of knee flexion. The outside‐in technique requires a femoral incision and more specialised equipment than the other two methods, including a backward drill. However, it has fewer restrictions on the placement of the femoral tunnel and allows for easier location of the tunnel due to the guidewire's position relative to the lateral supracondylar line [9].

**Figure 5:**
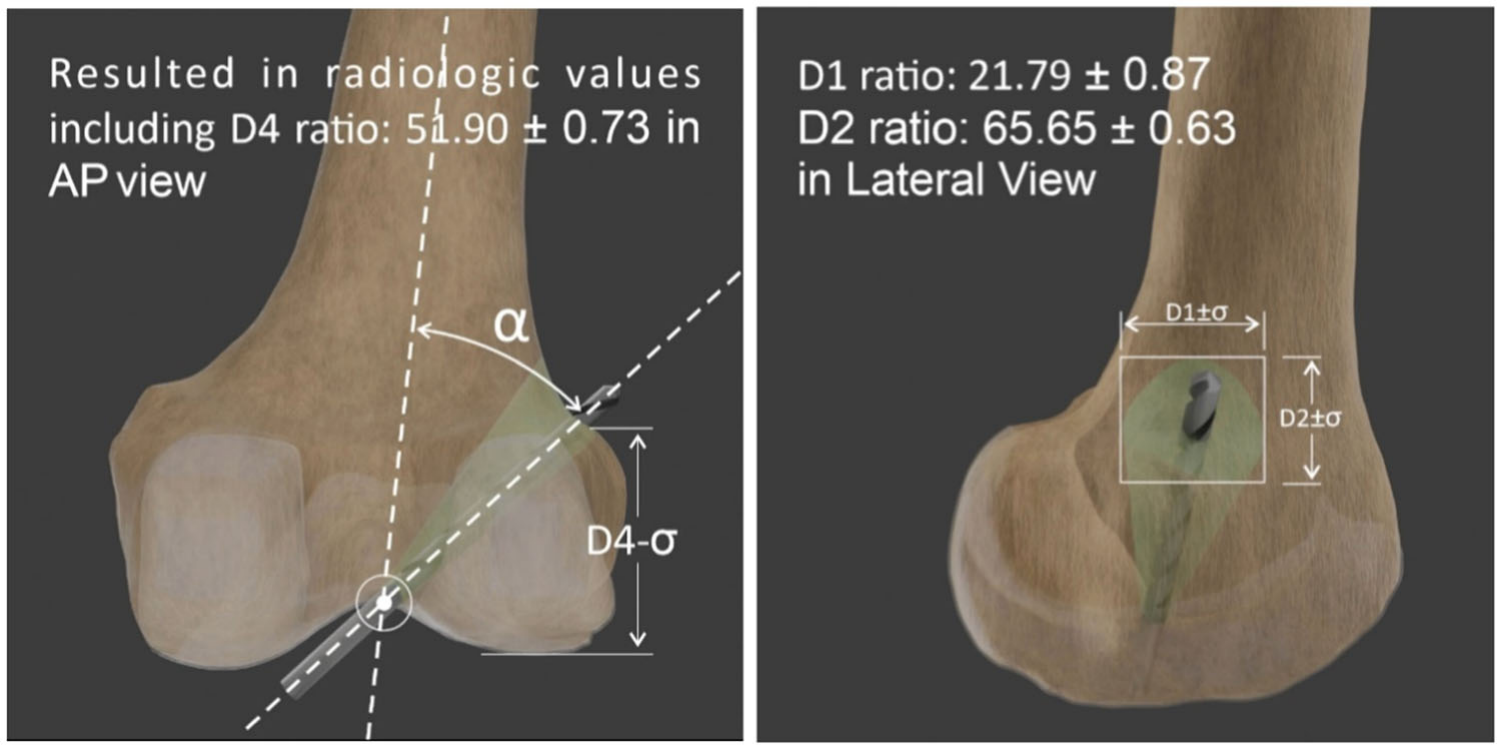
Safe zone according to suggested parameters.

To evaluate the accuracy of tunnel placement, three‐dimensional imaging such as computed tomographic scan can provide an accurate position of the tunnel, but due to high amount of radiation received in this method, intraoperative fluoroscopy and arthroscopic evaluation have been suggested as suitable options to assess the accuracy of tunnel placement during ACL reconstruction [12, 14]. Postoperative radiography also is a safe and inexpensive method for evaluation of endobutton location. In our study, the mean measured values of the anatomic parameters in X‐ray radiographs were found to be: D1 = 13.20 ± 0.54 mm, D2 = 39.44 ± 0.31 mm, D3 = 1.65 ± 0.15 mm and D4 = 42.66 ± 0.47 mm. The calculated ratios were D1 Ratio = 21.79 ± 0.87, D2 Ratio = 65.65 ± 0.63, D3 Ratio = 1.86 ± 0.17 and D4 Ratio = 51.90 ± 0.73 (Figure 5 and Video). Based on the current findings, the outlet site of the femoral tunnel during ACL reconstruction with the transportal technique includes a wide range, which aligns with previous studies. In the study by Gunaydin et al., which evaluated the effect of endobutton position on postoperative function, the lateral side of the femur was divided into three equal parts: anterior, middle and posterior. They found that the endobutton had a relatively uniform distribution across all three sections, with no significant difference in functional outcomes among these groups of endobutton positions [5].

Another finding of the study is the relationship between BMI and both D3 and the D3 ratio. An increase of 0.329 kg/m² in BMI corresponds to a 1 mm increase in D3, while a 0.355 kg/m² increase in BMI is associated with a 1 mm increase in the D3 ratio. Previous studies have mentioned body mass as a factor affecting femoral canal characteristics. According to Tompkins et al., height and BMI are positively related to tunnel length and negatively correlated with knee flexion angle. These factors can cause variation in the femoral tunnel outlet location [15]. In addition to the proven effect of body mass on the length and location of the femoral tunnel, the direct correlation between BMI and D3, which is the distance from the button centre to the lateral femoral cortex, might be attributed to the presence of soft tissue in this space. The interposition of soft tissue has been shown to have no effect on the long‐term outcome of ACL reconstruction [11].

Based on the study's findings, age was positively related to D2, which is the distance from the centre of the button to the line drawn vertically to the long axis of the femur at the distal‐most point of the lateral condyle. Although no studies have specifically examined the relationship between endobutton location and age, other research measuring tunnel‐related parameters such as length, widening and obliquity in relation to age has produced conflicting results [2, 3, 16, 19]. In our study, the relationship between age and D2 was controlled by calculating the D2 ratio, which considers the diameter of the femoral condyle. This adjustment accounted for the effect of femoral condyle size on the measured distance.

Considering the femoral tunnel length, a short tunnel length has been identified as a potential complication of the transportal technique due to the more horizontal placement of the tunnel within the femur, compared to the transtibial method [9]. In our study, the average canal length was 38.6 ± 0.2 mm, which is slightly longer than the values reported for patients undergoing ACL reconstruction with other techniques, typically ranging from 34 to 37 mm [6, 15, 17]. This unexpected result may be attributed to our efforts to maintain maximum knee flexion angle in our patients. Basdekis et al. have demonstrated the relationship between knee flexion angle and tunnel length, reporting a mean femoral tunnel length of 27.1 mm at 90° flexion, 38.9 mm at 110°, 38.8 mm at 130° and 39.2 mm at maximal flexion [1].

The present study has some limitations, including the exclusion of clinical evaluation and the assessment of factors such as knee size on the location of the endobutton. Consequently, further studies with inter‐ and intraobserver reliability evaluation are required to examine the correlation between appropriate endobutton location and the clinical outcome of ACL reconstruction. Additionally, these studies should assess the impact of other factors, such as knee size and the precise degree of knee flexion, on the endobutton location.

To minimise errors caused by changes in the imaging angle, the images were taken perpendicular to the body. Additionally, a sensitivity analysis of plane rotation was conducted to assess the remaining error and the impact of each degree of rotation. The relevant calculations will be attached. According to these calculations, deviations up to 3.5° do not significantly affect the measurement of the variables.

## CONCLUSION

In this study, a new approach was presented to assess the accuracy of anatomical tunnel placement in following ACL reconstruction surgery. The statistical analysis of the measured variables indicated that if the tunnel exit location and endobutton placement in the postoperative radiological images fall within the suggested areas, it can be meaningfully concluded that the tunnel is correctly positioned and the ligament reconstruction has been successful.

## AUTHOR CONTRIBUTIONS

**Arash Sharafatvaziri**: Conceptualisation; supervision; methodology; visualisation; data curation. **Mohammad Tahami**: Methodology; investigation; data curation. **Maryam Salimi**: Writing—original draft; writing—review and editing. **Hamid Rabie**: Writing—original draft; data collection; formal analysis. **Morad Karimpour**: Visualisation; investigation. **Fardis Vosoughi**: Writing—review and editing; formal analysis. **Ghazaleh Moradkhani**: Writing—review and editing. **Mosayeb Soleymani**: Project administration; methodology; investigation; writing—review and editing. All authors have read and approved the final manuscript.

## CONFLICT OF INTEREST STATEMENT

The authors declare no conflicts of interest.

## ETHICS STATEMENT

Written informed consent was obtained from the individuals in our study. The purpose of this research was completely explained to the patients, and they were assured that their information would be kept confidential by the researcher. All procedures performed in studies involving human participants were in accordance with the ethical standards of the institutional and/or national research committee and with the 1964 Helsinki Declaration and its later amendments or comparable ethical standards. Approval was granted by the ethical committee of Tehran University of Medical Sciences. Written informed consent was obtained from all individual participants included in the study. A copy of the written consent is available for review by the editor of this journal. Written informed consent was obtained from the patients regarding the publication of this study. There is no identifying information in this article.

## Supporting information

Supporting information.

## Data Availability

SPSS data of the participant can be requested from the authors. Please write to the corresponding author if you are interested in such data.
